# Ribociclib Hybrid Lipid–Polymer Nanoparticle Preparation and Characterization for Cancer Treatment

**DOI:** 10.3390/polym15132844

**Published:** 2023-06-28

**Authors:** Ramadan Al-Shdefat, Mohammad Hailat, Osama Y. Alshogran, Wael Abu Dayyih, Ahmed Gardouh, Osaid Al Meanazel

**Affiliations:** 1 Department of Pharmacy, Faculty of Pharmacy, Jadara University, Irbid 21110, Jordan; ahmed.ga@jadara.edu.jo; 2 College of Pharmacy, Al-Zaytoonah University of Jordan, Amman 11733, Jordan; m.hailat@zuj.edu.jo; 3 Department of Clinical Pharmacy, Faculty of Pharmacy, Jordan University of Science and Technology, Irbid 22110, Jordan; oyalshogran@just.edu.jo; 4 Faculty of Pharmacy, Mutah University, Al-Karak 61710, Jordan; wabudayyih@mutah.edu.jo; 5 Department of Pharmaceutics and Industrial Pharmacy, Faculty of Pharmacy, Suez Canal University, Ismailia 41522, Egypt; 6 Michael Sayegh Faculty of Pharmacy, Aqaba University of Technology, Aqaba 77110, Jordan; otalmeanazel@aut.edu.jo

**Keywords:** ribociclib, PLGA, hybrid nanoparticles, double emulsion method, pharmacokinetics

## Abstract

Ribociclib is a newly approved orally administered drug for breast cancer. This study aimed to prepare, characterize, and evaluate hybrid lipid–polymer nanoparticles (PLNs) of ribociclib to enhance its in vitro dissolution rate, pharmacokinetics, and anticancer efficacy. Ribociclib-loaded PLNs were prepared by solvent evaporation using the Box–Behnken design to optimize formulation variables. Particle size, entrapment efficiency, differential scanning calorimetry (DSC), thermogravimetric analysis (TGA), Fourier transform infrared spectroscopy (FTIR), atomic force microscopy (AFM), in vitro release cytotoxicity, molecular modeling, and pharmacokinetic studies were examined. The ribociclib-loaded PLN (formula 1, F1) was optimized in terms of particle size (266.9 ± 4.61 nm) and encapsulation efficiency (59.1 ± 2.57 mg/mL). DSC and thermogravimetric characterization showed the absence of a crystalline structure in the prepared PLNs, confirmed by FTIR, and showed no interactions between the components and the drug. AFM showed well-dispersed heterogeneously shaped nanoparticles. The in vitro release profile exhibited significant results for the optimized formula, reaching 100% at 600 and 90 min at pH 6.8 and 1.2, respectively. The low IC_50_ obtained by the 3-(4,5-dimethylthiazol-2-yl)-2,5-diphenyl-2H-tetrazolium bromide (MTT) assay suggests that optimized PLN might serve as an effective delivery vehicle for cancer treatment, especially breast and lung cancer. Molecular modeling revealed several hydrogen bonds. A pharmacokinetic study in rats showed that the ribociclib formula had a 6.5-fold increase in maximum concentration (C_max_) and a 5.6-fold increase in area under the curve (AUC). Regarding the everted intestinal sac absorption, formula 1 increased ribociclib penetration relative to the physical combination and pure medication. In conclusion, optimized PLNs with enhanced physicochemical and cytotoxic properties and improved pharmacokinetic parameters were successfully prepared.

## 1. Introduction

Cancer is the leading cause of mortality worldwide. As of 2022, the United States is expected to have 1,918,030 new cancer cases and 609,360 cancer deaths [1]. Traditional cancer treatments have come a long way, yet they still fall short of entirely curing patients. Incorporating many nanoparticles into a single formulation is an approach that nanotechnology can adopt to combat cancer. Liposomes and polymeric nanoparticles are the most studied and successful drug delivery systems (DDSs) for cancer treatment [2]. Liposomes are promising DDSs because of their desirable characteristics, such as high drug entrapment efficiency, biocompatibility, low cost, and scalability. Limited surface modification, sensitivity to lipid peroxidation, instability, and burst drug release limit their usefulness [3].

Similarly, polymeric nanoparticles exhibit several chemical changes with polymers, high stability, and controlled release; nevertheless, their limitations in drug loading, polymer toxicity, and scalability make them unsuitable for biological applications. Combining polymeric nanoparticles and liposomes forms hybrid lipid–polymer nanoparticles (PLNs). These PLNs benefit from both components’ advantages, including increased biocompatibility and stability, better drug payload, controlled drug release, increased circulation time, and increased in vivo efficacy [3].

In conjunction with an aromatase inhibitor, the FDA authorized ribociclib (as ribociclib succinate) in March 2017 to treat several types of breast cancer [4,5]. For post-menopausal women with hormone receptor-positive, HER2-negative, advanced or metastatic breast cancer, this drug combined with fulvestrant may provide an additional therapeutic option. Ribociclib is a small-molecule kinase inhibitor that selectively blocks CDK4 and CDK6 activities in the body [6]. Six hundred micrograms per day for the first 21 days of a 28-day cycle is the starting dose for the lower rate of corrected QT (QTc) prolongation with this dosing regimen [7].

Ribociclib is a crystalline powder with a molecular weight of 434.55 and is available as a succinate salt. Succinate salts have pKa values between 5.3 and 8.5. Its limited solubility in neutral solutions and intermediate permeability make it difficult to achieve appropriate bioavailability because it is a BCS class IV drug [8,9]. The water solubility of the drug decreases as pH increases from 2.0 and 7.5. As ribociclib has been authorized, the nanocarrier system has not yet addressed this issue. Fei and Yoosefian developed ribociclib micelles utilizing dodecylphosphocholine micelles to improve hydrophobic medication water solubility [10]. Another study examined the explicit creation and assessment of nanostructured lipid carriers created by solvent evaporation and administered ribociclib to address chemotherapeutic medication bioavailability issues in breast cancer [11].

As previously indicated, ribociclib solubility improves with decreasing pH and is unaffected by meal consumption or the pH of gastric contents [12]. A 1% m/V solution of succinate salt in distilled water has a pH of 5.19. Aqueous water solubility in acidic conditions is about 2.4 mg/mL, but in neutral conditions, ribociclib succinate suffers from low water solubility, and the free base solubility is 0.63 mg/mL [8,9]. Cytochrome P450 3A4 extensively breaks down ribociclib. Thus, cytochrome P450 3A4 modulators are affected and have limited efflux transport to the brain by cytochromes. In contrast, high ribociclib exposure has been linked to an increased risk of neutropenia [8,12,13].

This manuscript reports the creation and assessment of novel nanostructured lipid carriers created by solvent evaporation and orally administered to address issues related to medication bioavailability. This study aimed to prepare optimized ribociclib hybrid lipid–polymer nanoparticles using PLGA as a biodegradable polymer via the double emulsion solvent evaporation technique. The experimental plan used Box–Behnken statistical designs to generate 34 runs and determine the independent variables influencing the model’s dependent components and fitness. The independent variables could be changed in our study (polymer type and concentration, lipid concentration, and surfactant concentration). In contrast, the dependent variables were those that we determined in our study for the prepared formulae (particle size, loading capacity, and zeta potential). The optimized formulation characterization and evaluation included particle size, polydispersity index (PDI), zeta potential measurements, loading capacity of ribociclib polymer nanoparticles, thermal characterization of ribociclib nanoparticles, X-ray diffraction (XRD), Fourier transform infrared spectroscopy (FTIR), in vitro release using a dialysis bag approach, atomic force microscopy (AFM) evaluation, everted intestinal sac for ex vivo evaluation of absorption, cytotoxicity studies using the MTT assay, pharmacokinetic studies in rats, and molecular modeling. The results showed promising drug loading and formulation using tristearin, with particle sizes ranging from 64 to 520 nm and a polydispersity index of less than one.

## 2. Materials and Methods

### 2.1. Materials

The company that supplied us with ribociclib was Beijing Mesochem Technology Company (Beijing Mesochem Technology Company, Beijing, China). Poly(D,L-lactide-co-glycolide) 50:50 (RESOMER^®^ RG 502 S 0.16-0.24) and poly(D,L-lactide-co-glycolide) 75:25 (RESOMER^®^ RG 752 S 0.16-0.24) were purchased from Evonik Operations GmbH (Darmstadt, Germany). Kolliphor^®^ P188, soy phospholipids, glyceryl tristearate, and dialysis membrane (MW = 12,000 Da) were purchased from Sigma-Aldrich (Software (St. Louis, MO, USA)). Merck KGaA supplied ethyl acetate (Darmstadt, Germany). A Milli-Q^®^ system was used to purify the water (Millipore, Bedford, MA, USA). Acetonitrile, methanol, and high-performance liquid chromatography (HPLC) grade water were obtained from Fisher Scientific. Formic acid was purchased from Merck. All other reagents and chemicals used were of analytical grade.

### 2.2. Methods

#### 2.2.1. Plans for Conducting Experiments to Improve the Formula

Box–Behnken statistical designs were used to optimize the formula. The independent variables were the type and concentration of PLGA, lipid concentration, and surfactant concentration, whereas the dependent variables were the particle size, zeta potential, and loading capacity, as listed in Supplementary Table S2. The Design-Expert^®^ (version 11.0.0; Stat-Ease Inc., Minneapolis, MN, USA) application built an experimental plan using a quadratic polynomial equation (quadratic model). Design-Expert^®^ software is a statistical application designed specifically for design of experiment (DOE) procedures. DOE is a scientific approach for conducting experiments to identify factors that influence a process or product efficiently and effectively. Design-Expert^®^ software is well known for the design of experiments in various scientific fields, especially dosage form design, where we can assume independent variables that we can execute and also the responses from which the program, utilizing probability theory, will design different trials that we can execute with concentration ranges that we control, resulting in our case in 34 formulae. We concluded the research with advanced characteristics for samples selected based on the optimal size, zeta potential, and loading capacity.

A total of 34 formulae were generated using Design-Expert^®^ software in response to input variables, such as polymer type and concentration, surfactant concentration, and lipid concentration, as well as proposed responses, such as particle size, zeta potential, and loading capacity. These preparations established various relationships between the input variables and the proposed responses. Based on our findings, we determined that F1, F17, and F25 had the highest loading capacities, zeta potentials, and smallest particle sizes. Therefore, these samples were selected for this study (Table 1). PLGA was chosen based on previous studies (10, 20, and 30 mg/mL of organic phase), and glyceryl tristearate lipids (5, 10, and 15 mg/mL of organic phase) were chosen based on rough scanning of different surfactants with 0.5, 1, and 1.5% *w*/*v*. Another factor that was considered was the polymer type, which affected the experimental responses. The major components of the polymer–lipid nanoparticulate system include drugs (10 mg), polymers, lipids, and surfactants [14,15,16].

Analysis of variance was used to determine the independent variables influencing the model’s dependent components and fitness. All batches of nanoparticles were tested statistically (*p* < 0.05).

#### 2.2.2. Preparation of the Ribociclib-Loaded PLNs

**Scheme 1:**
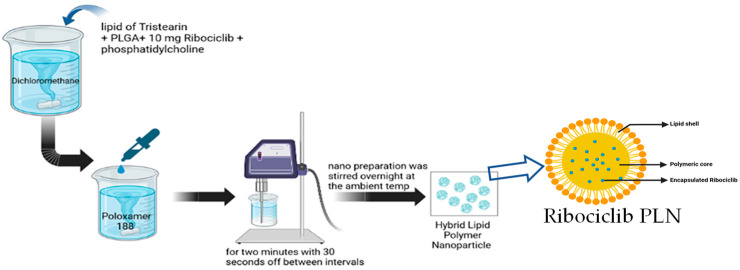
**Preparation of ribociclib-loaded PLNs:** Tristearin lipid, the biodegradable polymer poly(D,L-lactide-co-glycolide) (PLGA, 50:50 or 75:25 grades), ribociclib (10 mg), and 30 mg phosphatidylcholine were dissolved in 3 mL of DCM. This organic solution was added dropwise to the aqueous phase containing P188 with continuous magnetic stirring, and a probe ultrasonicator emulsified the two phases for two minutes with 30-s breaks. The nanopreparation was agitated overnight to eliminate the organic solvent.

Ribociclib lipid–polymer nanoparticles were created using the method described by Al-Thamarani et al. (2021) [17], with slight modifications [17]. The lipid tristearin, biodegradable polymer poly(D,L-lactide-co-glycolide) (PLGA, 50:50 or 75:25 grades), and ribociclib (10 mg) mixed with 30 mg phosphatidylcholine were dissolved in 3 mL of dichloromethane (DCM, organic phase). This organic solution was added dropwise to an aqueous phase containing poloxamer 188 under continuous stirring (magnetic stirrer). The two phases were emulsified using a probe ultrasonicator for two minutes at 30 s intervals. The nanopreparation was stirred overnight under ambient conditions to remove organic solvent [14] (Scheme 1) [14].

#### 2.2.3. The Optimized Formulation’s Characterization and Evaluation

##### Particle Size, Polydispersity Index (PDI), and Zeta Potential (ζ)

The average particle size, particle distribution index (PDI), and zeta potential of the combined PLNs were determined using a DelsaMax Pro Analyzer (B29164, Beckman Coulter, Brea, CA, USA). Triple PLN size, PDI, and zeta potential measurements were taken after injecting materials into “folded capillary cells”.

##### Loading Capacity of Ribociclib Polymer Nanoparticles

The samples were centrifuged using a freeze ultracentrifuge (Mx series, Himac CP 100 MX, Hitachi, Tokyo, Japan) at 15,000 rpm for 15 min, and the supernatant was collected to determine the free drug. The entrapment efficiency (EE%) of ribociclib was determined by HPLC of the supernatant after centrifugation of the polymeric nanoparticle suspension and calculated using Equation (1) [18,19].

(1) $$EE\%=\frac{\left(W_{\mathrm{total}}-W_{\mathrm{free}}\right)}{\left(W_{\mathrm{total}}\right)}\times 100\%$$

where W_total_ = weight of the drug initially added to the formulation. W_free_ is the amount of free drug in the supernatant.

#### 2.2.4. Thermal Characterizations of Ribociclib Polymer Nanoparticles: Differential Scanning Calorimetry (DSC) and Thermogravimetric Analysis (TGA)

Thermal analysis of ribociclib nanoparticles was performed for ribociclib, PLGA, tristearin, the physical mixture, and ribociclib-loaded PLNs. Thermograms were obtained using a differential scanning calorimeter (DSC NETZSCH, DSC 204 F1 Phoenix, NETZSCH, Selb, Germany) and thermogravimetric analyzer (TGA, MODEL TG 209 F1 Iris, NETZSCH, Selb, Germany). Ten micrograms of the sample was placed in an aluminum pan and scanned at 10 °C/min between 25 and 350 °C under nitrogen gas [20]. Melting point peaks and drug and/or additive degradations were also evaluated.

#### 2.2.5. X-ray Diffraction (XRD)

X-ray diffraction (XRD) was performed using a Miniflex 600 (Rigaku, Tokyo, Japan) at 40 kV and 20 mA in the 2θ range of 10–80° at a scan rate of 3.0000 ° min^−1^ to explore the amorphous and crystalline characteristics of the drug and formulae.

#### 2.2.6. Fourier Transform Infrared Spectroscopy (FTIR)

FTIR is an essential complementary tool for the solid-state characterization of pharmaceutical solids [21]. Physical or chemical interactions between the drug and other formulation components were identified. An FTIR spectrometer (FTIR SHIMADZU, Kyoto, Japan) was used to obtain FTIR spectra of ribociclib, polymers, lipids, and the physical mixtures of drugs, lipids, and polymers forming nanoparticles (model IRAffinity-1, SHIMADZU, Kyoto, Japan). Spectra were obtained at a resolution of 4 cm^−1^ with 16 scans per sample over a 4000–400 cm^−1^ range.

#### 2.2.7. In Vitro Release

The in vitro release profile of ribociclib was obtained for PLNs using the dialysis bag approach [22]. The dialysis bag (molecular weight cutoff 12,000–14,000) was immersed in deionized water for 24 h [23]. The cellulose bag was immersed in sodium phosphate buffer (pH 6.8) for two hours, simulating the intestinal fluid release profile at 37 °C ± 0.2 after being filled with 1 mL of nanoparticle suspension while knotted at both ends (samples of 1 mL withdrawn at 15, 30, 60, 90, and 120 min and replaced with fresh medium). Samples of 1 mL were collected at predetermined intervals (30, 60, 90, 120, 180, 240, 360, 480, 600, and 720 min) to measure the ribociclib release profile and replaced with 1 mL of fresh medium to maintain sink conditions.

The concentration of ribociclib was determined using a modified HPLC method published earlier [24]. The cumulative percentage of drug released was examined in triplicate. The formulation that exhibited the best drug release was investigated.

#### 2.2.8. Atomic Force Microscopic (AFM) Evaluation

AFM samples were generated by distributing the nanoparticle solution over a newly sliced mica (grade II) substrate and drying the solvent with a nitrogen stream. For AFM analysis, the samples were measured at ambient temperature. Non-contact AFM was performed using a Smart SPM 1000 scanning probe microscope with a controller (AIST-NT, Novato, CA, USA). The AFM tips were model NSCM/A1BS etched Si tips with a resonance frequency of 160 kHz. AFM images were collected from a PLN nanostructured film produced by vertical adsorption from a colloidal solution onto glass plates for 5 min and then dried in air. Silver colloid and 0.01 M anesthetic solutions were mixed in equal volumes using the same method.

#### 2.2.9. Everted Intestinal Sac for Ex Vivo Evaluation of Absorption

The jejunum and ileum were dissected from the gut (5–6 cm each). Cold oxygenated Krebs solution was used to wash the sections (pH 6.5). A 1 mL micropipette was used to inject 1 mL of normal saline at 37 °C into the everted end of the cleaned intestine draped over a glass rod and a braided silk suture was used to secure the filled intestinal segment. At 37 °C, 1 milligram (mg) of ribociclib in 25 milliliters (mL) of the oxygenated medium was added to the everted filled sac, and the entire object was placed in an incubation flask. Samples were collected every 15 min (i.e., every 15, 30, 45, and 60 min) and then every 90 and 120 min from the start of the study [25].

#### 2.2.10. Cytotoxicity Studies (MTT Assay)

The treatments were assayed for cell toxicity using an MTT assay. Cytotoxicity was evaluated by observing cell survival in the culture. Six serial dilutions (200, 100, 50, 25, 12.5, and 6.2 µg/mL) were used to fit the calculations. We used an Excel add-in on the website [26] to calculate IC50 [27]. MTT assays were performed using the optimized formula F1 and the parent drug. For this experiment, cells were plated at a density of 1 × 10^4^ cells/well in 96-well plates using a multichannel micropipette into 8 wells in triplicate at each dilution point and incubated in DMEM at 37 °C for 24 h before being switched to DMEM containing various dosages of treatments for another 48 h [28]. The medium was discarded, and the cells in each well were treated with 20 µL of MTT solution (5 mg/mL) for 4 h at 37 °C as part of the MTT assay. The MTT solution was discarded, and 200 µL of dimethyl sulfoxide (DMSO) was added to dissolve the insoluble formazan crystals. Optical densities were determined at 570 and 630 nm. Data were collected from eight independent wells. Fibroblasts isolated from human periodontal ligaments (PDLs) were utilized to confirm selective cytotoxicity with the lowest antiproliferative IC_50_ value. The IC_50_ antiproliferative activities were determined by performing experiments in triplicate and reporting the mean SD (*n* = 3).

##### Cancer Cell Lines

The breast cancer cell line MDA-MB-231 (mammary gland/breast) was acquired from a metastatic site, pleural effusion, and the pancreatic cancer cell line PANC-1 (ATCC CRL1469) (PDL). All cells were cultured in DMEM supplemented with 10% fetal bovine serum, 10 mM HEPES buffer, 100 µg/mL of L-glutamine, 50 µg/mL of gentamicin, 100 µg/mL of penicillin, and 100 mg/mL of streptomycin.

All the cell cultures were maintained at 37 °C in a humidified incubator with 5% CO_2_. After washing the cells in 3–5 mL of phosphate-buffered saline (PBS), the cells in 75 cm^2^ flasks were detached by adding 1–2 mL of trypsin to each flask. Each cell line had the same volume of new media added and was then pipetted gently to break any clumps and create a homogenous single-cell suspension. There was a noticeable variation across cell lines concerning the frequency and ratio of cell proliferation. After achieving the required quantity, cells were multiplied every two–three days. One hundred microliters of trypan blue dye (4% concentration) was combined with 25 µL of the extracted cells, and the resulting cell suspension was placed at the edge of the chamber for counting.

##### Cytotoxicity Studies (MTT Assay) of the Formulated Nanoparticles

The cytotoxicity of the formed nanoparticles of ribociclib and a physical combination of solvent components was evaluated. Cytotoxicity was evaluated by observing cell survival in the culture. We used six serial dilutions (200, 100, 50, 25, 12.5, and 6.2 µg/mL) to fit these calculations. We used an Excel add-in on the website [26] to calculate the IC50. MTT assays were performed using the optimized formula F1 and the parent drug. Cells were plated at a density of 1 × 10^4^ cells/well in 96-well plates and cultured for 24 h at 37 °C in DMEM before being switched to a medium containing various treatment doses for an additional 48 h. After draining the medium, 20 µL of MTT solution (5 mg/mL) was added to each well, and the cells were incubated at 37 °C for 4 h. After removing the MTT solution, 200 µL of dimethyl sulfoxide (DMSO) was added to dissolve the insoluble formazan crystals. Optical densities were determined at 570 and 630 nm. Data were collected from three independent wells. To confirm selective cytotoxicity with the lowest antiproliferative IC_50_ value, human periodontal ligament fibroblasts (PDLs) were used as the primary cell culture. Three independent replicates of each experiment were conducted, and the mean ± standard deviation (*n* = 3) of the IC_50_ antiproliferative activity was reported.

#### 2.2.11. Pharmacokinetic Study in Rats

The Animal Care Facility at the Jordan University of Science and Technology (JUST), Jordan, provided ten male Wistar albino rats weighing 200–250 g. The animals were accommodated for 1 week in the animal facility under hygienic conditions at 22–25 °C with a 12 h light/dark cycle. The rats were housed in steel cages and provided regular meals and access to water at all times. Rats were randomly divided into two groups (*n* = 5 per group). The first group received 5 mg/kg ribociclib powder dispersed homogeneously in 0.1% carboxymethylcellulose (CMC) solution, whereas the second group received a 5 mg/kg ribociclib nanoparticle formulation. The diet was removed before administering the medication for 8–10 h while having free access to water. Ribociclib and the nanoparticle formulation were administered as a single dose via oral gavage. Blood samples were collected from the retro-orbital plexus vein into heparinized tubes at the following time points: 0.00, 0.25, 0.5, 1, 1.5, 2, 4, 8, and 24 h after drug administration. The collected samples were centrifuged at 4500 rpm for 12 min to obtain plasma and stored at −80 °C until further analysis. The Animal Care and Use Committee (ACUC) at JUST (16/4/12/409), which follows the National Institutes of Health Standard for the Care and Use of Laboratory Animals, approved the research protocol (NIH Publication No. 8023).

Ribociclib concentration in plasma was analyzed using liquid chromatography–tandem mass spectrometry (LC-MS/MS). Highly purified water containing 0.1% formic acid and acetonitrile containing 0.1% formic acid in a ratio of 15:85 (*v*/*v*) comprised mobile phases A and B, respectively. The column was maintained at a steady temperature of 25 °C. A constant flow rate of 0.4 mL/min throughout the sample processing. After 10 min, every sample was eluted [29].

Phoenix WinNonlin was used to derive the pharmacokinetic parameters from the known plasma concentrations of ribociclib. Using non-compartmental pharmacokinetic analysis, we determined the maximum plasma concentration (C_max_), time to maximum concentration (T_max_), terminal elimination half-life (t_1/2_), the area under the concentration–time plot from zero to the last measurable concentration (AUC_last_), and t_1/2_ (MRT). We compared the means of the two groups using the Student’s *t*-test at a statistical significance level of *p* < 0.05. The latest version of GraphPad Prism was used for statistical analyses.

#### 2.2.12. Molecular Modeling

##### Preparation of Synthesized Compounds

The goal of molecular docking is to predict the binding posture of a ligand in a protein’s active site. To treat adults with hormone receptor (HR)-positive, human epidermal growth factor 2 (HER2)-negative breast cancer, the 2D chemical structure of the standard inhibitor (ribociclib) was saved as a mol file in ChemDraw Ultra (Version 11.0.1), converted to a 3D structure, and minimized to the closest local energy minimum using the CHARMm force field in CATALYST. The production of ribociclib conformers had an upper limit of 250 kcal/mol and an energy threshold of 20 kcal/mol relative to the closest local minimum. Using the rule-based conformational procedures in DS 7.6, the 2D structure was transformed into a 3D structure and stored in the SD for docking studies.

##### Preparation of HER2 Structure

The Protein Data Bank (PDB) entry for HER2 (3RCD, resolution: 3.21 A°) containing the molecule’s three-dimensional structure was used. Hydrogen atoms were incorporated into the protein using DS 7.6 protein residue templates. Within a DS of 7.6, atomic charges were assigned using the Gasteiger–Marsili method for protein molecules. In 1980, Gasteiger and Marsili reported that no further energy reduction in the protein structure was performed before docking trials.

##### LibDock Docking

Like the binding hotspots used in previous studies, LibDock guides ligands to their proper binding sites [30].

The current docking experiments employed specific parameters for the docking and scoring of ribociclib (Supplementary Material).

## 3. Results and Discussion

This study investigated the influence of different formulation compositions on characteristic formulations. The particle size, PDI, ZP, and % drug encapsulation of the manufactured PLNs were evaluated. The novel formulations were subjected to a series of tests, including morphological analysis, in vitro release studies, in vitro MTT assays against breast cancer cell lines, and molecular modeling docking.

### 3.1. Particle Size, Loading Efficiency, and Zeta Potential of Prepared Formulae

**Figure 1:**
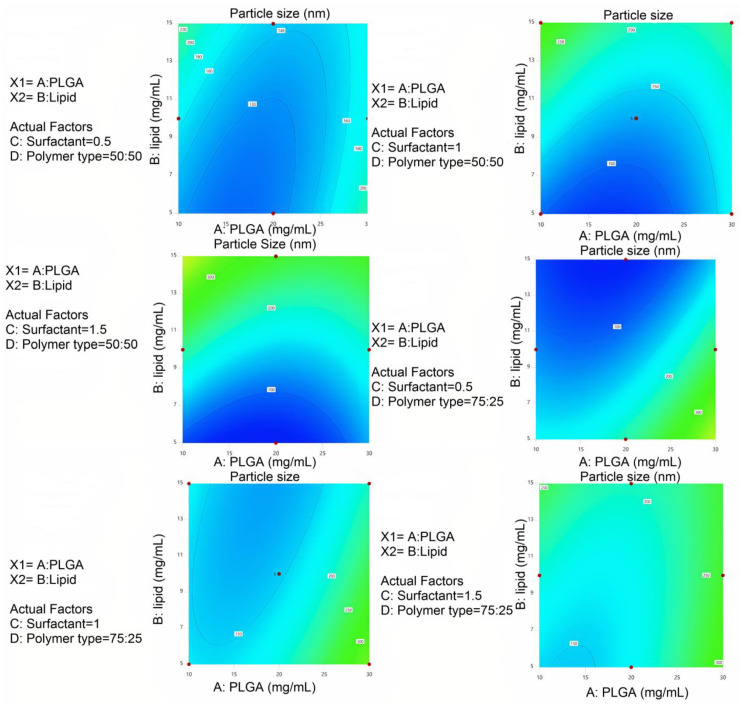
The 3D plots for the relationship between PLGA type and concentration and tristearin concentration on the size of hybrid polymeric nanoparticle formulae at different poloxamer 188 concentrations.

**Figure 2:**
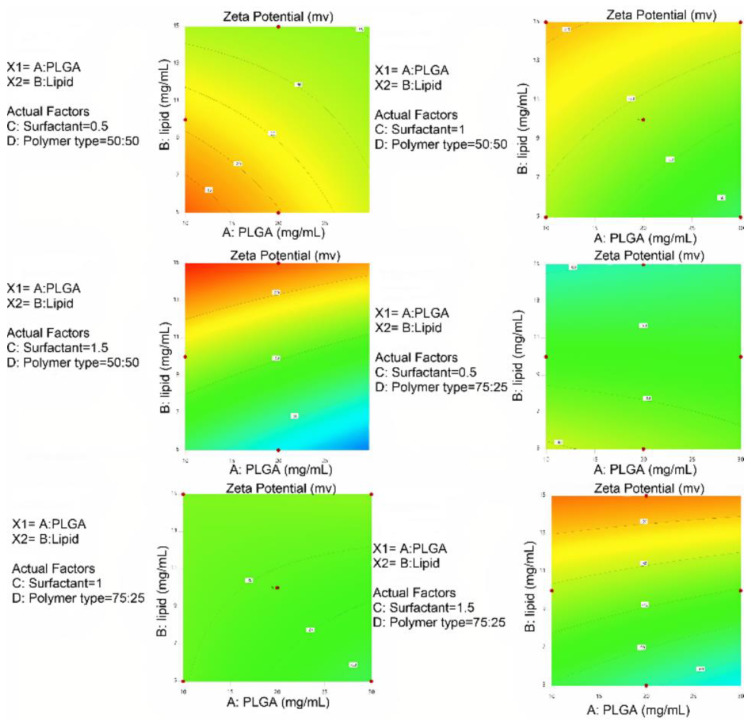
The 3D plots of the relationship between PLGA type and concentration and tristearin concentration on the zeta potential of hybrid polymeric nanoparticle formulae at different poloxamer 188 concentrations.

**Figure 3:**
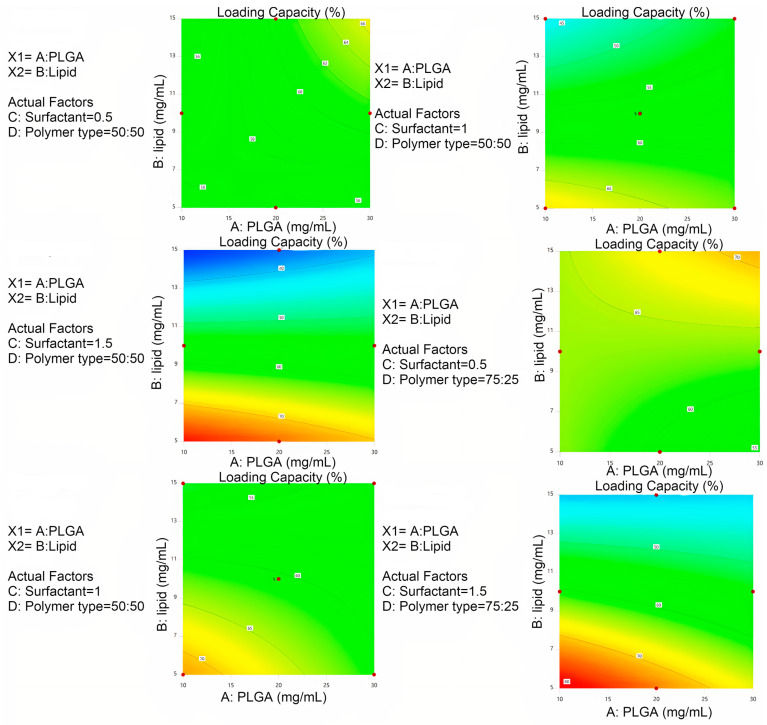
The 3D plots for the relationship between PLGA type and concentration and tristearin concentration on the loading capacity percent of hybrid polymeric nanoparticle formulae at different poloxamer 188 concentrations.

The quadratic polynomial generated using the Box–Behnken design and Design-Expert^®^ 7.0.0 software was used to establish a correlation between the various factors and formulations. The particle sizes ranged from 64 to 520 nm. These findings are consistent with previous research that used a modified nanoprecipitation technique to create hybrid PLNs [31]. The polydispersity index of all the prepared formulae determined by the laser diffraction technique was less than one, indicating homogeneity and a normal distribution. The entrapment efficiency of the PLNs ranged from 38.7% to 79.8%. The acquired ζ potential (from −2.61 to −69.59 mV) confirmed the stability and particle homogeneity of the formulated products. Figure 1, Figure 2 and Figure 3 show the particle size, loading capacity, and zeta potential of the ribociclib-loaded PLNs. In the pre-formulation stage, tristearin was tested with different lipids, such as glyceryl monostearate, distearate, and tristearate, indicating promising results related to drug loading and formulation using tristearin as a lipid. (Further data are available and can be found in the Supplementary Information, Supplementary Tables S1 and S2.)

#### 3.1.1. Effect of Factor X1:PLGA 50:50 Concentration

As PLGA concentration increased, the size of the mixed PLNs also increased. Figure 1, Figure 2 and Figure 3 demonstrate that when the PLGA concentration was elevated, the size of the nanoparticles increased, which is consistent with the results of Chan et al. [32]. Larger emulsion droplets are produced because nanoparticle production is suppressed by the high-viscosity solution resistance and highly concentrated solution aggregates [33,34,35]. The ζ potential of the mixed PLNs increased with increasing PLGA content, and Chan et al. [32] revealed a growing trend in the NP size with a corresponding increase in ζ potential. Supplementary Tables S1 and S2 show that as the PLGA concentration increased, the size of the mixed PLNs’ EE increased, which is consistent with the results of Xie et al. [36]. When the PLGA concentration was increased, the encapsulation efficiency of all formulations improved dramatically. Drug leakage was reduced, and encapsulation improved due to the relatively high polymer concentration during production [37].

#### 3.1.2. Effect of Factor X2: Tristearin (TS)

The size of PLNs increased as the tristearin concentration increased [34]. Figure 1, Figure 2 and Figure 3 suggest that the deposition of many tristearin layers caused nanoparticle expansion. The enhanced ζ potential of the mixed PLNs was due to elevated lipid (TS) content. Similar results have been reported by Kongtong et al. [38]. According to the study findings, forming a lipid-coated PLN surface increased the concentration of the negative charge, which is similar to the findings of Mancini et al. [39], who used a lipid material to create solid lipid NPs with a negative surface charge.

#### 3.1.3. Effect of Factor X3: Poloxamer 188

Figure 1, Figure 2 and Figure 3 show that when the concentration of poloxamer 188 was increased, the overall movement of the response surface shifted from significant to smaller [34,40,41]. The model graphs indicate this. Santander-Ortega et al. discovered that PLGA particles exhibited negative mobility [42]. Even with Poloxamer 188, the particles retained their negative mobilities.

However, when the surfactant load increased, the mobility decreased. As the surfactant concentration increases, it has been demonstrated that structural changes in the adsorbed poloxamer chains cause variations in electrokinetic activity. The results showed that the ζ potential of the PLGA particles was reduced owing to the screening effect of the absorbed non-ionic poloxamer coating. When the surfactant concentration (0.5% *w*/*v*) was reduced, the overall movement of the entrapment efficacy decreased, indicating that an insignificant quantity of the surface-active agent was necessary to establish a stable colloidal system, as illustrated in Supplementary Tables S1 and S2. Furthermore, more significant surfactant concentrations (1.5% *w*/*v*) resulted in smaller particle sizes and more significant entrapment owing to the solubilizing effect of surfactants and decreased interfacial tension [43]. (Additional information is available in the Supplementary Materials).

Consequently, as shown in Supplementary Table S1, formula 1 was used for further characterization, including TEM, pharmacokinetic, and pharmacodynamic studies.

### 3.2. In Vitro Release of Ribociclib from Prepared Formulae

**Figure 4:**
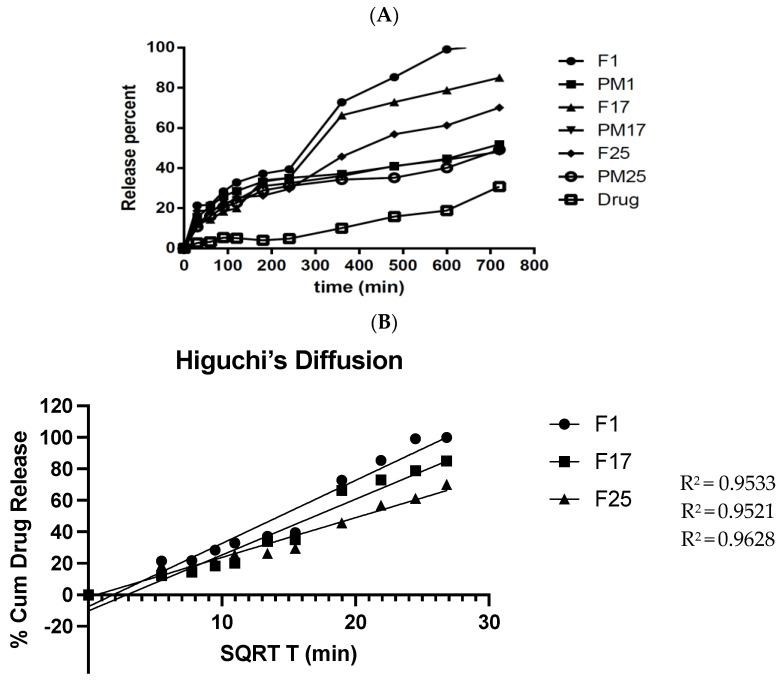
(**A**) In vitro drug release (cumulative release percentage in mg versus time in min) at pH 6.8. Formula number 1 (F1), physical mixture for formula 1 (PM1) (which is composed of 30 mg/mL of PLGA of type 75:25, 10 mg/mL lipid, and 1.5 mg/mL surfactant), formula number 17 (F17), physical mixture for formula 17 (PM17) (which is composed of 10 mg/mL of PLGA of type 50:50, 5 mg/mL lipid, and 1 mg/mL surfactant), formula number 25 (F25), physical mixture of formula 25 (PM25) (which is composed of 10 mg/mL of PLGA of type 75:25, 5 mg/mL lipid, and 1 mg/mL surfactant), and the drug (ribociclib), (**B**) Higuchi’s model showed the best fit of the release data for F1, F17, and F25 formulae.

The in vitro release profile of optimized formulations with the desired particle size, zeta potential, entrapment efficiency, mixed PLN equivalent to the formulated formulation (F1), and pure ribociclib was studied using a cellulose membrane as a semi-permeable membrane. After 6 h at pH 6.8, the mixed PLN formulations, F1, and pure ribociclib, released 40%, 100%, and 20%, respectively (Figure 4A). In addition, formula 1 failed to produce an initial burst release, suggesting complete encapsulation in the carrier matrix and a lack of unbound free drug concentrations on the surface of the nanoparticles, followed by a continuous release, which is in agreement with the results of Omwoyo et al. [44]. The rate of drug release was highest in the F1 formula.

Mathematical models help create pharmaceutical formulations, analyze drug release mechanisms, and optimize system designs. They measure the drug diffusion coefficient and fit the experimental release data to predict the quantity and kind of active agent, polymer, adjuvants, and system size and shape [45]. Our in vitro release data were fitted into a variety of common kinetic models, including zero order, first order, Higuchi, Hixson–Crowell, and the Korsmeyer and Peppas model that uses cube root law for cube root percentage of drug remaining vs. time.

Table 2 shows the correlation coefficients of the kinetic models used to fit the release of ribociclib from the formulations at pH 6.8. The Higuchi kinetic model was chosen as the best model that describes the release of ribociclib since it had the highest R^2^ values compared to other applied kinetic models. Higuchi fits the experimental release data shown in Figure 4B.

Higuchi’s 1961 equation described the release rate of drugs from matrix systems, specifically ointment bases [46]. His 1962 analysis of data on drug release from ointments and 1963 proposal of the “mechanism of sustained-action medication” further advanced the understanding of drug release in soluble and non-soluble active agents [47,48]. In Higuchi’s model, the amount of drug released is proportional to the square root of time, that is:

$$F=K_{h}\;\times \;\sqrt{t}$$

where F: fraction of drug released at time t, K_h_: Higuchi release rate constant.

The model equations of ribociclib formulae were as follows: F1: Y = 4.004 × X − 7.426; F17: Y = 3.545 × X − 10.16; F25: Y = 2.524 × X − 1.505. The highest slope (release rate constant) was for F1 (Kh = 4.004), which indicates the fastest release of ribociclib from F1 as compared to other formulae. This could be explained by the smaller particle size and lower PDI values for F1 versus other formulae.

Equation (1) is the simplified Higuchi model, which represents a linear function by relating the concentration of active agents to the square root of time. The Higuchi model requires the following assumptions: (1) the initial drug concentration in the matrix is substantially higher than the drug’s solubility. (2) As the edge effects are negligible, the diffusion is unidirectional. (3) The dosage form’s thickness is substantially more than the size of the medication molecules. (4) The matrix’s swelling or disintegration is insignificant. (5) The drug’s diffusivity is constant. (6) In the release environment, optimal sink conditions are reached. Higuchi’s models facilitated the development of several critical mathematical methodologies in classifying the potential release profile of active compounds in dosage forms. As a result, as shown in Table 2 and Figure 4, nanoparticle drug release followed the Higuchi order release kinetic model. These findings may be consistent with the findings of Shoaib et al. (2006), who reported that drug release followed the Higuchi kinetic model [49].

### 3.3. Characterization of Ribociclib PLNs

#### 3.3.1. DSC

DSC thermal analysis measures the heat capacity of a material as a function of the temperature. DSC can identify nanoparticle interactions by measuring the melting point, heat of fusion, and thermal conductivity and is a powerful tool for investigating the crystallinity of nanoparticles. DSC tests were performed to validate compatibility. The thermal behavior of drugs, polymers, physical mixtures, and drug-loaded nanoparticles was investigated. Any significant changes in the thermal behavior of drugs or polymers may indicate drug–polymer and lipid interactions [50]. The heating curves from DSC are shown as enthalpy (*m*/*w*) vs. temperature (°C) plots [51].

**Figure 5:**
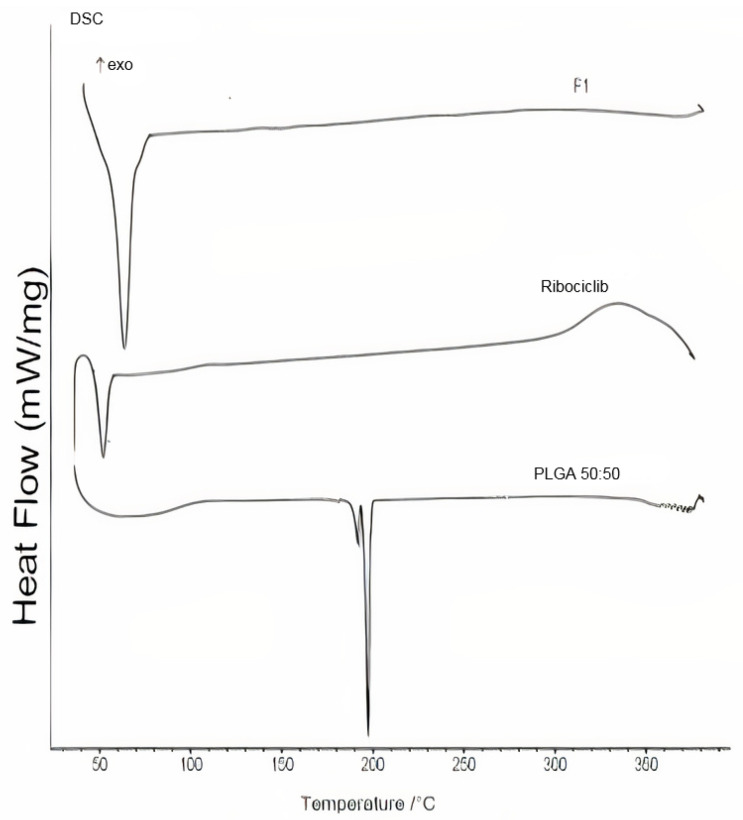
DSC thermographs (heat flow versus temperature in degrees Celsius) of ribociclib, PLGA 50:50, and formula 1 (F1), which were used for better visualization.

Ribociclib dissolved in the polymer–lipid matrix, diminishing its characteristic crystallinity but retaining its therapeutic activity, as seen by the shifting of the endothermic peak of both ribociclib and PLGA 50:50 and the changes in its intensity [52], as shown in Figure 5.

#### 3.3.2. XRD

**Figure 6:**
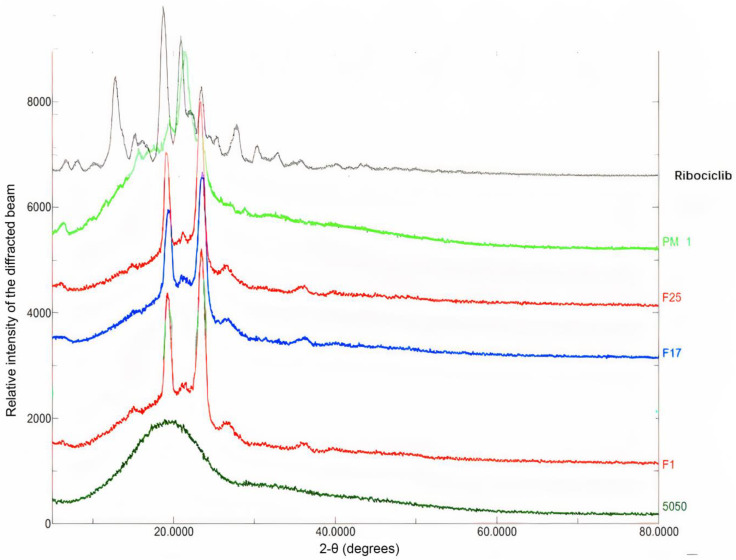
XRD (intensity versus diffraction angle) for ribociclib, physical mixture for formula 1 (PM 1, composed of 30 mg/mL PLGA of type 75:25, 10 mg/mL lipid, and 1.5 mg/mL surfactant), formula number 25 (F25), formula number 17 (F17), formula number 1 (F1), and a physical mixture of the components in a 50:50 (5050) mixture. As seen in the XRD figure, the distinctive peaks of the drug were still present in the physical combination, F1, F17, and F25, as well as the pure drug.

Powder X-ray diffraction investigations are critical for determining whether a powder is crystalline or amorphous. The XRD diffraction patterns of the physical mixtures of free ribociclib, F1, F17, and F25, shown in Figure 6, revealed several sharp peaks in pure ribociclib; however, these peaks vanished or were reduced in intensity in the ribociclib-loaded formulations, indicating that ribociclib could be encapsulated in PLNs.

#### 3.3.3. TGA

**Figure 7:**
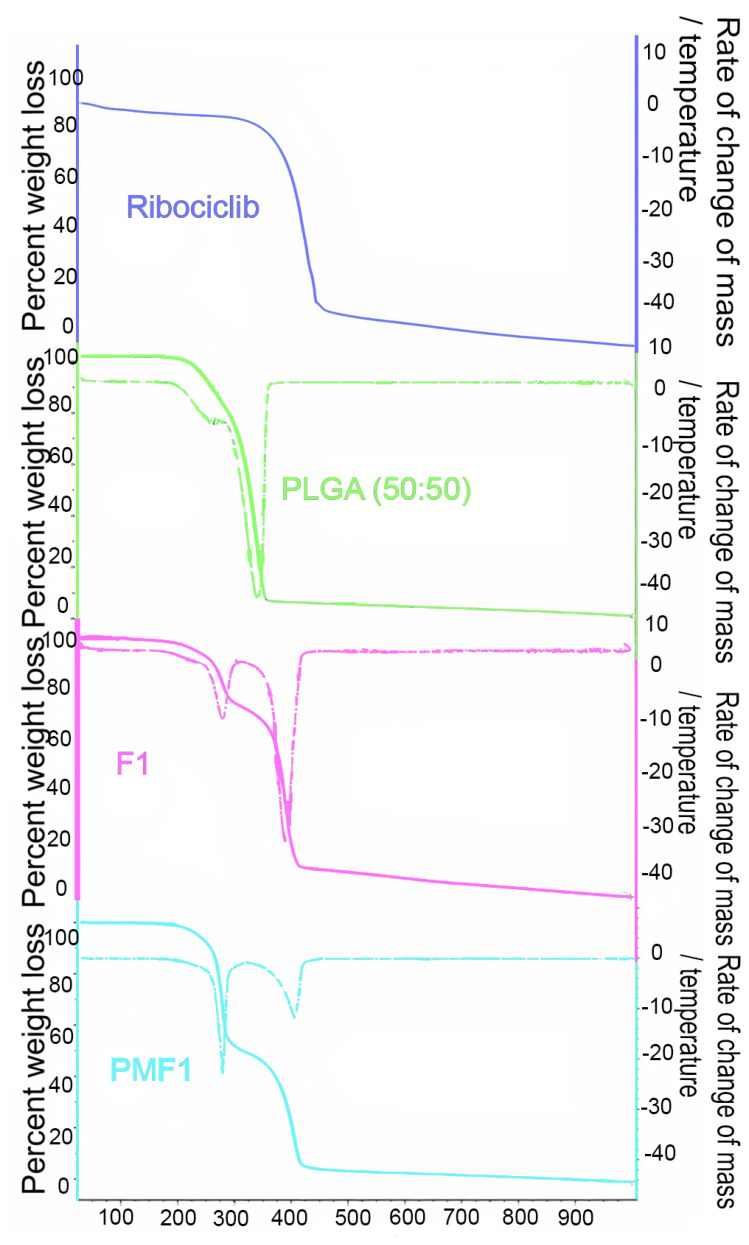
TGA thermographs (weight change or mass loss versus temperature in °C) of ribociclib, PLGA 50:50, physical mixture (PMF1), F1, F17, and F25, which are shown for better visualization.

TGA was used to characterize ribociclib and its optimized formula in the solid state. Figure 7 shows the TGA spectra of ribociclib and its optimized formula. The deterioration of ribociclib occurred between 671.2 and 723.2 K. TGA revealed no evidence of water loss between 298.2 and 523.2 K. TGA demonstrated that the optimized formulations were thermodynamically stable until approximately 400 °C.

#### 3.3.4. FTIR

**Figure 8:**
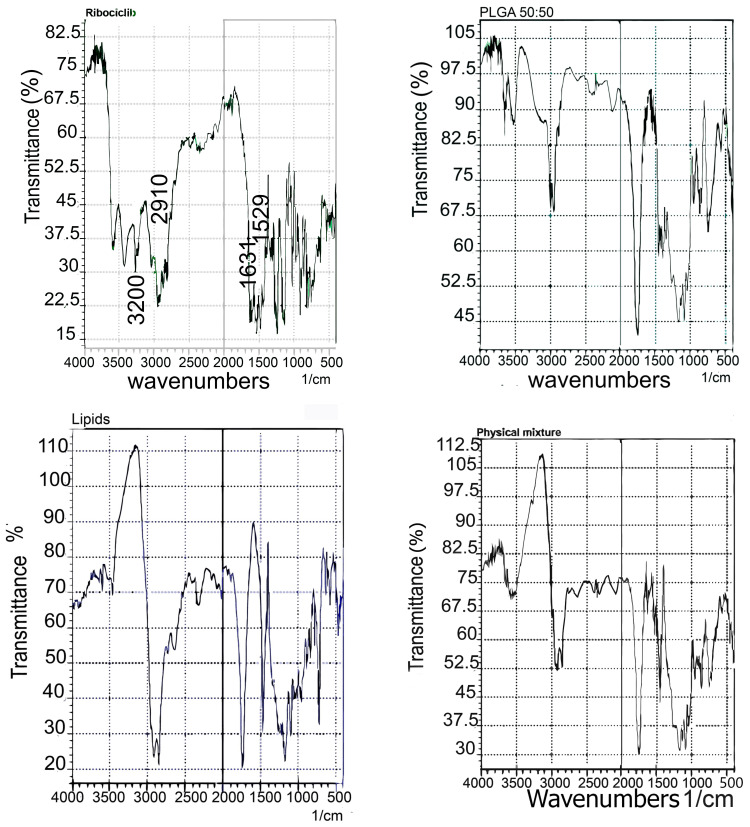

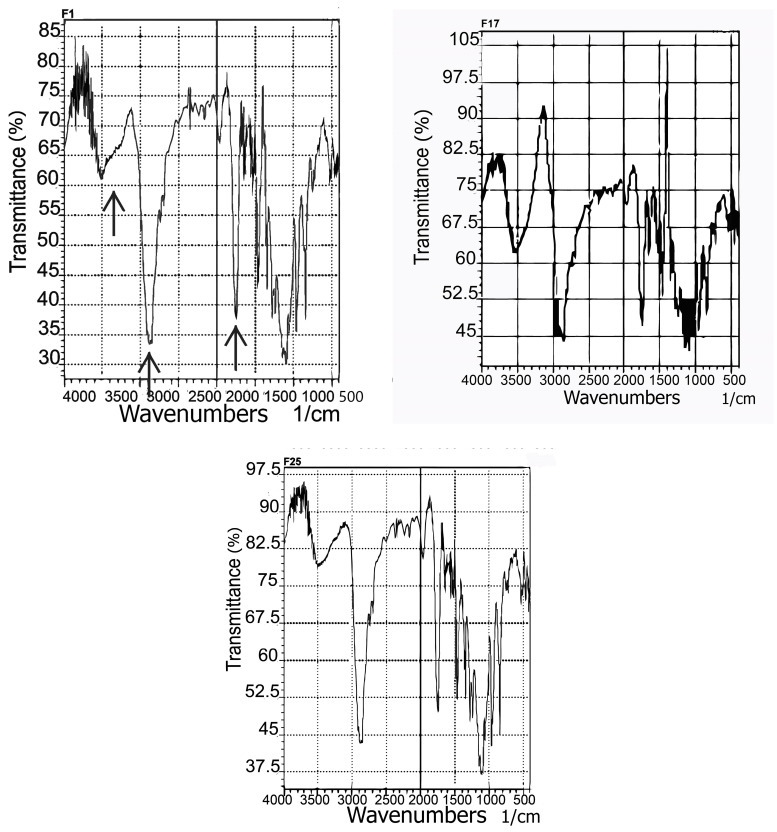
FTIR spectra (transmittance versus wavenumbers) of ribociclib, lipid, PLGA 50:50, physical mixture (PM), F1, F17, and F25, which are shown for better visualization.

Figure 8 shows typical NH stretching, C=H stretching, C=O stretching, and NH bending vibration peaks of pure ribociclib at 3200 cm^−1^, 2914 cm^−1^, 1631 cm^−1^, and 1529 cm^−1^, respectively. As the strength in the fingerprint area of ribociclib decreased, the results indicated that similar peaks in the spectrum shifted or disappeared [53]. This proves that the medication was physically enclosed inside the polymer matrix (Figure 8).

#### 3.3.5. AFM

**Figure 9:**
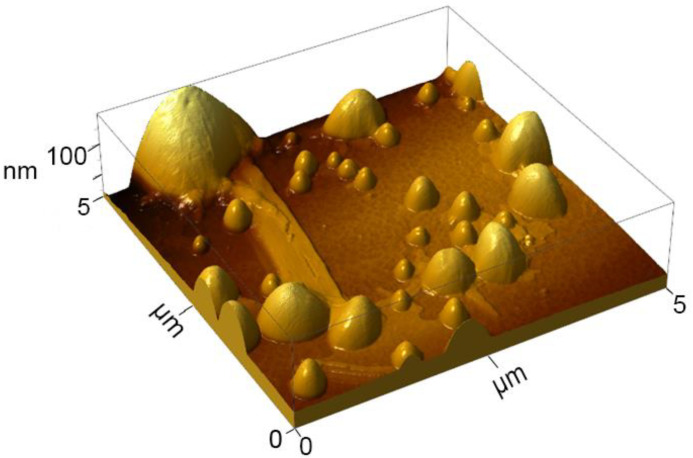
Atomic force microscopy images (height versus lateral position) of PLNs on the glass; scanned area 1 μm × 1 μm: 3D topography. The image shows well-dispersed heterogeneously shaped nanoparticles.

AFM was used to image a 3 µL solution of the NPs that had been air-dried on a clean mica surface. Figure 9 displays 5 × 5 µm topographical images and film surface contour plots in a cross-section through the PLN layer as an example of AFM images. While air-drying and surface interactions may affect the appearance of adsorbed particles to varying degrees, they appeared to be fluffy, with variable heights, smoothness, and ruffled edges. The AFM results showed that the PLNs, slightly polydisperse, had a spherical structure with a smooth surface and were 180–220 nm in size. The nanoparticles had an diameter of roughly 100 nm and a peak height of 70 nm. Due to drying and surface impact, the morphologies (aggregates) identified by AFM may differ from those in the solution.

#### 3.3.6. Everted Intestinal Sac for Ex Vivo Evaluation of Absorption

**Figure 10:**
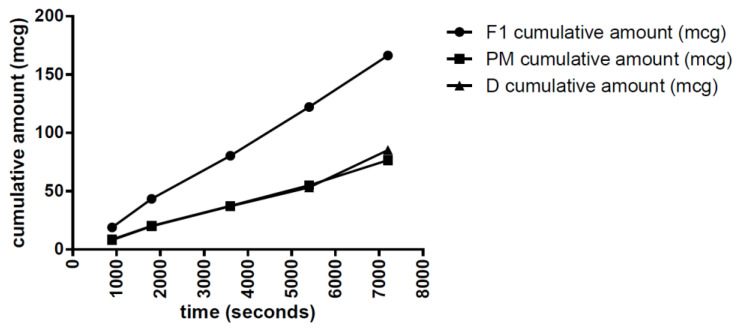
The cumulative amount of ribociclib permeated through everted intestinal sac absorption. F1: formula 1, PM: physical mixture; D: ribociclib.

The results were obtained from an everted intestinal sac calculated according to a previous study [54], where cumulative amounts of drug transport across rat intestinal segments were represented versus time, and the apparent permeability was calculated (Figure 10) [54]. When ribociclib permeation from formula 1 was compared to that of the physical mixture and pure drug, it was discovered that the nanoparticle size of formula 1 augmented drug permeation, whereas drug permeation from the physical mixture was nearly the same as that of the pure drug. As a result, formula F1 reached 166.4 mcg at 7200 s, whereas the physical mixture and pure drug reached 76.6 and 85.2 mcg, respectively.

#### 3.3.7. Cytotoxicity Studies (MTT assay)

The MTT assay revealed that pure medication and tailored-made PLNs reduced the viability of MDA-MB-231, pancreatic cancer cell line PANC-1, human lung cancer cell line A549, and human periodontal ligament (PDL) fibroblasts in a concentration-dependent manner. The IC50 values for MDA-MB-231 cells and optimized PLNs were 57.58 µg/mL and 4.34 µg/mL, respectively, for pancreatic cancer cell line PANC-1, 56.17 µg/mL, 46.6 µg/mL for human lung cancer cell line A549, and 56.36 µg/mL and 47.1 µg/mL for human periodontal ligament (PDL) fibroblasts. However, the optimized PLNs showed 84.5, 82.5, 78.5, and 73.5% inhibition at 200, 100, 50, and 25 µg/mL, respectively, compared to the pure drug (81.5, 80.5, 78.5, and 76.5% inhibition at 200, 100, 50, and 25 µg/mL) against MDA-MB-231 cells. In contrast, the optimized PLNs showed 91.5, 88.5, 85.5, and 70.5% inhibition at 200, 100, 50, and 25 µg/mL, respectively, compared to the pure drug (92.0, 91.0, 82.5, and 70.0% inhibition at 200, 100, 50, and 25 µg/mL) against the human lung cancer cell line A549 (Table 3).

Based on the MTT test findings, it was discovered that optimized PLNs demonstrated potential anticancer affinity against breast cancer cell lines and the human lung cancer cell line A549 as a consequence of enhanced drug release from optimized PLNs compared to pure drugs. Drugs loaded into optimized PLNs may be powerful carriers for breast and lung cancer therapies.

### 3.4. Pharmacokinetic Study in Rats

**Figure 11:**
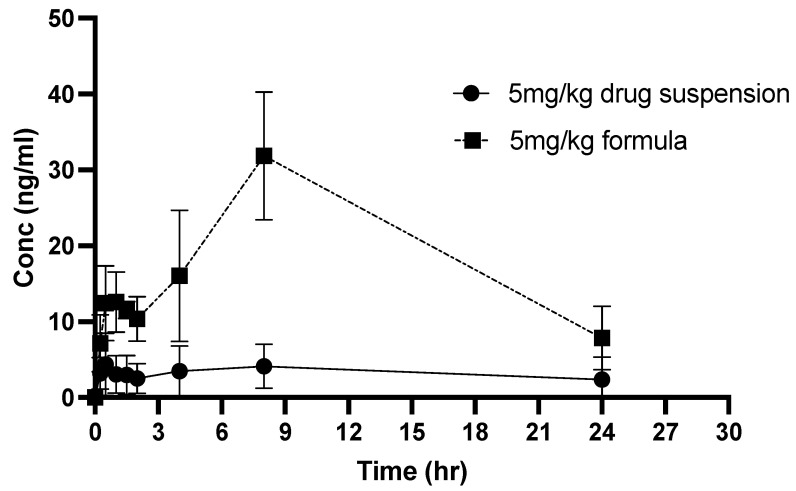
Plasma concentration–time curves of ribociclib suspensions or nanoparticle formulations in rats.

Figure 11 depicts the mean plasma concentration versus the time profile of ribociclib in rats after oral administration of the standard drug suspension and the developed nanoparticle formulation. The calculated PK parameters are listed in Table 4. Ribociclib exposure was significantly higher in rats administered the nanoparticle formula than in those administered the standard drug suspension, as the PLNs demonstrated a 6.5-fold increase in C_max_ and a 5.6-fold enhancement in AUC_last_ (*p* < 0.001). A 2.2-fold increase in the total AUC was observed with the nanoparticle formulation compared to the standard drug. Other PK parameters are shown in Table 4. Previous PK studies of ribociclib in various species have shown rapid oral absorption, moderate bioavailability, first-pass effect, extensive distribution, and high metabolism mediated primarily by CYP3A4 [53,54,55]. The PK data in this study revealed a remarkable increase in ribociclib plasma exposure with the optimized nanoparticle formulation. This could reflect enhanced relative oral bioavailability. The observation that the half-life was not different between the two groups suggests that increased bioavailability was related to enhanced absorption and/or reduced first-pass metabolism of the drug rather than decreased ribociclib elimination.

Data are presented as the mean ± SD (*n* = 5) for each group, except for T_max_, which is presented as the median (range).

The ribociclib concentration–time profiles in both groups showed a distinct double peak phenomenon. This intriguing finding warrants further investigation because it could be related to variable gastric emptying, multiple drug absorption sites in the intestine, or enterohepatic circulation [54,55].

### 3.5. Molecular Modeling

Supplementary Figure S1 shows the two-dimensional structure of ribociclib and the three-dimensional structure of docked ribociclib within the binding pocket of HER2 protein (PDB Code: 3RCD). The types and interaction forces are illustrated in Supplementary Figure S2A,B. Hydrophobic attractive forces include pi-sigma, alkyl, or pi-alkyl types between the aliphatic moieties of ribociclib such as cyclopentyl, purine ring, pyridine ring, and the methyl group of the amide side chain with different amino acids such as LEU 755, LEU796, LYS753, ALA751, PHE864, MET774, LEU785, and LEU852. The hydrogen atom attached to the carbon atoms of the piperazine aliphatic ring and the N-methyl group of the amide form carbon–hydrogen interactions with the oxygen atoms MET801 and GLY865, respectively. Hydrogen bonds were observed between the N atom of the pyrimidine ring as a hydrogen bond acceptor and THR862 as a hydrogen bond donor. Finally, the pi bonds of the purine ring formed π-atom interactions with ASP863. The amino acids were involved in the interaction forces within the binding pocket of the HER2 protein structure. This could be used to explore the binding site and its nature for the selection and synthesis of additional HER2 inhibitors.

## 4. Conclusions

PLNs were successfully prepared using PLGA as a biodegradable polymer via double emulsion solvent evaporation. The prepared nanoparticles, optimized using the Box–Behnken design response surface method, showed enhanced release from the formulations, colloidal stability, and high loading capacity, with better in vitro and simulated in vivo release of the drug. The pharmacokinetic parameters showed better absorption and higher C_max_ of ribociclib from the prepared nanoparticles. Compared with the standard drug suspension, the coated nanoparticle formulation of ribociclib demonstrated improved oral bioavailability, suggesting that the prepared PLNs are a helpful tool for delivering low-solubility drugs, such as ribociclib. Such PLNs have been proposed as a promising drug delivery system for future prospects of drug delivery to provide greater absorption and higher C_max_ for cancer treatment.

## Figures and Tables

**Table 1:** Components of Formulae (F) 1, 17, and 25.

Formula	PLGA Type	PLGA Conc. (mg/mL)	Tristearin Conc. (mg/mL)	Kolliphor^®^ P188 Conc. (%*w*/*v*)
F1	75:25	30	10	1.5
F17	50:50	10	5	1
F25	75:25	10	5	1

**Table 2:** Values of correlation coefficients for various fitting models of ribociclib release from the formulae at pH 6.8.

Formula	Correlation Coefficients (r)				
	Zero Order	First Order	Higuchi’s Diffusion Model	Hixson–Crowell Model	Korsmeyer–Peppas
F 1	0.953	0.850	0.953	0.944	0.927
F 17	0.950	0.892	0.952	0.910	0.960
F 25	0.952	0.980	0.963	0.973	0.904

**Table 3:** Cytotoxicity studies (MTT assay). IC_50_ results of MTT assay using formula 1 for breast cancer, fibroblasts, lung cancer, and PANC-1 cell lines.

Cell Type	IC_50_ for Optimized F1	IC_50_ for the Parent Drug
Breast cancer	4.3	57.6
Fibroblasts	47.1	56.4
Lung cancer	16.1	51.7
PANC-1	46.6	56.2

**Table 4:** Pharmacokinetic parameters after oral administration of a 5 mg/kg ribociclib suspension or nanoparticle formulation in rats.

Parameter	Unit	5 mg/kg Drug Suspension ± SD	5 mg/kg Formula ± SD
C_max_	ng/mL	6.48 ± 3.7	31.85 ± 8.4 *
T_max_	h	4 ± 0.25	8 ± 0.37 *
AUC_last_	ng * h/mL	81.66 ± 6.61	460.74 ± 12.61 *
T_1/2_	h	31.2 ± 3.0 *	7.84 ± 2.04
MRT	h	9.38 ± 1.4	9.18 ± 0.9

* Indicates a significant difference between the two groups (*p* < 0.05) using an unpaired Student’s *t*-test.

## Data Availability

The data presented in this study are available in this article.

## Supplementary Materials

The following supporting information can be downloaded at: https://www.mdpi.com/article/10.3390/polym15132844/s1, Figure S1: (A) 2D structure of co-crystalline compound Ribociclib against (B) 3D structure of Ribociclib; Figure S2: (A) Interaction forces of Ribociclib within the binding pocket of HER2 protein (PDB Code: 3RCD) (B) 2D presentation of Interaction forces of Ribociclib within the binding pocket of HER2 protein (PDB Code: 3RCD); Table S1: The results of different formulas preparation; Table S2: Variables used in design expert process.
