# Supplementary material for: Ribociclib Hybrid Lipid–Polymer Nanoparticle Preparation and Characterization for Cancer Treatment
Source: Polymers (Basel). 2023 Jun 28;15(13):2844. doi: 10.3390/polym15132844 (PMC10347058; doi:10.3390/polym15132844)
Supplement: Supplementary file 1 [file polymers-15-02844-s001.zip › polymers-2411642-supplementary.pdf]

Supplementary Table S1: The results of different formulas preparation.

formulation number	PS± SD (nm)	ZP ± SD (mV)	LC ± SD (mg/mL)
1	266.9 ± 4.61	-52.98 ± 2.30	59.1 ± 2.57
2	520 ± 22.61	-68.29 ± 2.97	63.2 ± 2.75
3	87.2 ± 3.79	-62.11 ± 2.70	70.3 ± 3.06
4	173.9 ± 7.56	-69.56 ± 3.02	60.5 ± 2.63
5	145.2 ± 6.31	-45.91 ± 2.00	79.8 ± 3.47
6	57.5 ± 2.50	-89.81 ± 3.91	59.5 ± 2.59
7	55.1 ± 2.40	-60.98 ± 2.65	80 ± 3.48
8	126.9 ± 5.52	-58.64 ± 2.55	74.7 ± 3.25
9	64 ± 2.78	-54.20 ± 2.36	64.5 ± 2.80
10	208.3 ± 9.06	-32.84 ± 1.43	60.3 ± 2.62
11	100.3 ± 4.36	-54.77 ± 2.38	60.9 ± 2.65
12	188.3 ± 8.19	-34.25 ± 1.49	60.4 ± 2.63
13	314.5 ± 13.68	-30.60 ± 1.33	44.2 ± 1.92
14	95.5 ± 4.15	-46.56 ± 2.02	38.7 ± 1.68
15	149.6 ± 6.51	-23.70 ± 1.03	48.7 ± 2.12
16	127.3 ± 5.54	-40.41 ± 1.76	56.7 ± 2.47
17	104.6 ± 4.55	-43.22 ± 1.88	72.1 ± 3.14
18	253.6 ± 11.03	-11.09 ± 0.48	59.4 ± 2.58
19	179.5 ± 7.81	-32.45 ± 1.41	57.6 ± 2.50
20	184.3 ± 8.01	-11.59 ± 0.50	31 ± 1.35
21	184.5 ± 8.02	-8.46 ± 0.37	58.7 ± 2.55
22	263.6 ± 11.46	-2.61 ± 0.11	44.8 ± 1.95
23	178.6 ± 7.77	-19.01 ± 0.83	62.6 ± 2.72
24	107.1 ± 4.66	-41.80 ± 1.82	65.9 ± 2.87
25	138 ± 6.00	-41.42 ± 1.80	78.7 ± 3.42
26	164.1 ± 7.14	-11.26 ± 0.49	562 ± 24.44
27	180.2 ± 7.84	-36.76 ± 1.60	45.2 ± 1.97
28	284.6 ± 12.38	-38.85 ± 1.69	67.3 ± 2.93
29	150.5 ± 6.54	-8.69 ± 0.38	48.2 ± 2.10
30	131 ± 5.70	-17.36 ± 0.75	512 ±2.26
31	275.7 ± 11.99	-29.18 ± 1.27	39.7± 1.73
32	135.3 ± 5.88	-11.71 ± 0.51	65 ± 2.83
33	120.7 ± 5.25	-23.88 ± 1.04	52.8 ± 2.30
34	176.7 ± 7.68	-41.61 ± 1.81	56.9 ± 2.47

Abbreviations: PS: particle size, ZP: zeta potential, LC: loading capacity.

Supplementary Table S2: Variables used in design expert process.

Formulation	PLGA (mg/mL)	Lipid (mg/mL)	Surfactant (w/v)
1	30[a]	10	1.5
2	30[a]	5	1
3	20[b]	5	1.5
4	10[a]	10	0.5
5	20[a]	10	1
6	30[b]	5	1
7	20[b]	10	1
8	20[b]	15	0.5
9	20[a]	10	1
10	30[a]	10	0.5
11	20[b]	15	0.5
12	20[a]	10	1
13	20[b]	15	1.5
14	20[a]	10	1
15	10[a]	15	1
16	20[b]	10	1
17	10[b]	5	1
18	20[b]	10	1
19	20[b]	10	1
20	20[a]	15	1.5
21	20[b]	5	0.5
22	10[b]	15	1
23	30[b]	10	0.5
24	20[a]	5	1.5
25	10[a]	5	1
26	30[b]	10	1.5
27	10[b]	10	1.5
28	10[a]	10	1.5
29	10[b]	10	0.5
30	20[b]	10	1
31	30[b]	15	1
32	20[a]	10	1
33	20[a]	5	0.5
34	30[a]	15	1

[a]: The ration of LA:GA is 75:25 in PLGA used.
[b]: The ration of LA:GA is 50:50 in PLGA used.

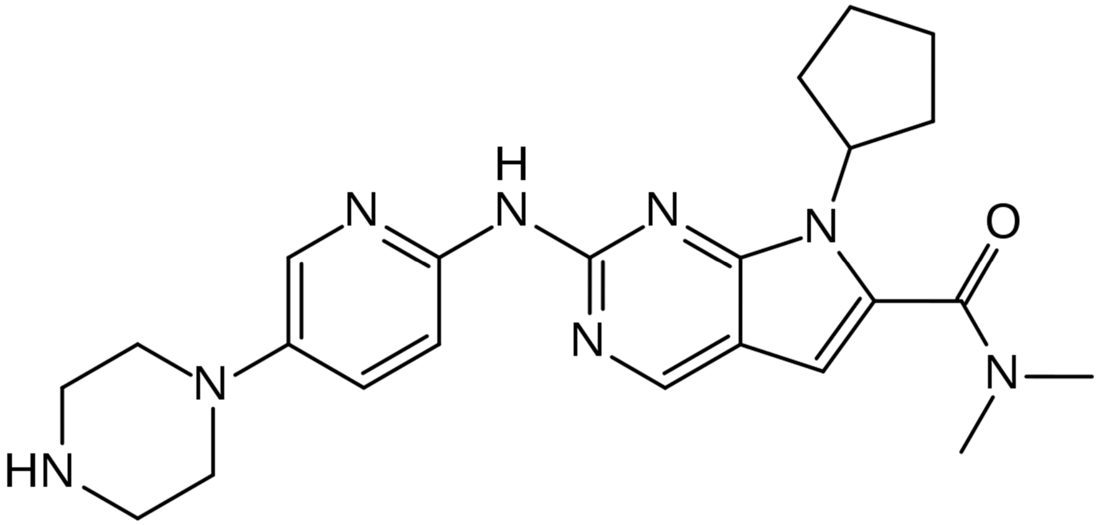

A

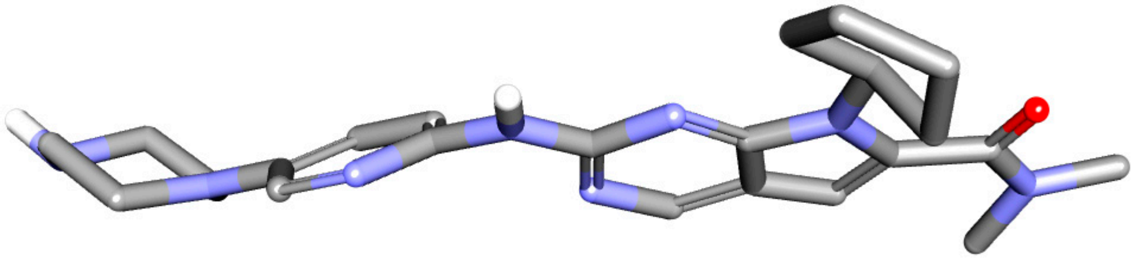

B

Supplementary Figure S1: (A) 2D structure of co-crystalline compound Ribociclib against (B) 3D structure of Ribociclib

LibDock Docking :

•Binding site dimensions of sphere are: 13.4562, 2.65794, 7.4454, 10.6374Å surrounding the center of the cocrystallized ligand (ORP) or Torcetrapib.

•Maximum number of receptor hotspots = 100.

• Docking Tolerance value was set to 0.25 Å.

•Maximum number of ligand saved poses = 100.

•Maximum number of poses saved for each ligand during hotspots matching before entering thefinal pose minimiza-tion = 100.

•Minimum LibDock score (poses below this score are not reported) = 100.

•Fraction of reported top scoring poses = 0.5.

•Maximum number of rigid body minimization steps during final pose optimization phase (using BFGS method) = 50.

•Maximum number of evaluated poses for each conformation = 30.

•Maximum number of steric clashes allowed before the pose-hotspot alignment is terminated (specified as a fraction of the heavy atom count) = 0.10.

•Cluster similarity cutoff value = 0.5 Å (docked poses are rigid-body minimized and clustered using this cutoff value).

•Maximum value for nonpolar solvent accessible surface area for a particular pose to be reported as successful= 15.0 Å2

.•Maximum value for polar solvent accessible solvent area fora particular pose to be reported as successful = 5.0 Å2.

•Number of grid points used for calculating solvent accessible surface area = 18.

•Conformation generation method: The CATALYST module CATCONFIRM implemented in DS 2.0 was implemented employing the BEST conformation generation option to ensure the best coverage of the compound's conformational space. Maximum number of conformations to be generated per ligand = 255 not exceeding an energy threshold of 20 kcal/mol from the most stable conformer. No final ligand minimization was implemented (i.e., in the binding pocket).

•The docked poses were scored employing the same seven scoring functions that were implemented in LigandFit docking experiment and employing identical parameters.

Input ligands: 14
Conformers generated: 3109
Poses docked: 1396

Input Site Sphere	10.1156, 1.45628, 37.8399, 8.26768
Protocol Settings	Protocol.pr xml
Input Receptor	4ews.dsv
Input Ligands	Manal chemicals with Activity.sd

Input Site Sphere	10.1156, 1.45628, 37.8399, 8.26768
Number of Hotspots	100
Docking Tolerance	0.25
⊟ Docking Preferences	High Quality
Max Hits to Save	100
Max Number of Hits	100
Minimum LibDockScore	100
Final Score Cutoff	0.5
Max BFGS Steps	50
Rigid Optimization	False
Keep Hydrogens	False
Max Conformation Hits	30
Max Start Conformations	1000
Steric Fraction	0.10
Final Cluster Radius	0.5
Apolar SASA Cutoff	15.0
Polar SASA Cutoff	5.0
Surface Grid Steps	18
⊟ Conformation Method	BEST
Maximum Conformations	255
Discard Existing Conformations	True
Energy Threshold	20.0
DSReport Summary	False
Separate Conformations	False
⊟ Minimization Algorithm	Do not minimize
RMSD Cutoff	1.0
Flexible Residues	
Minimization Max Steps	1000

Minimization RMS Gradient	0.001
Minimization Energy Change	0.0
Minimization Forcefield	CHARMm
Minimization Sphere of Flexible Atoms	
Implicit Solvent Model	Distance-Dependent Dielectrics
Dielectric Constant	1
Implicit Solvent Dielectric Constant	80
Generalized Born Lambda Constant	
Minimum Hydrogen Radius	0.8
Use Non-polar Surface Area	True
Non-polar Surface Constant	0.92
Non-polar Surface Coefficient	0.00542
Salt Concentration	0.0
Input Atomic Radii	van der Waals radii
Use Molecular Surface	True
Nonbond List Radius	13.0
Nonbond Higher Cutoff Distance	12.0
Nonbond Lower Cutoff Distance	9.0
⊟ Advanced	
Verbose	0
sp2-sp2 rotation	True
Grid Scoring	True
Hotspots File	
⊟ Parallel Processing	True
Batch Size	25
Server	localhost

Server Processes 2
Preserve Order True

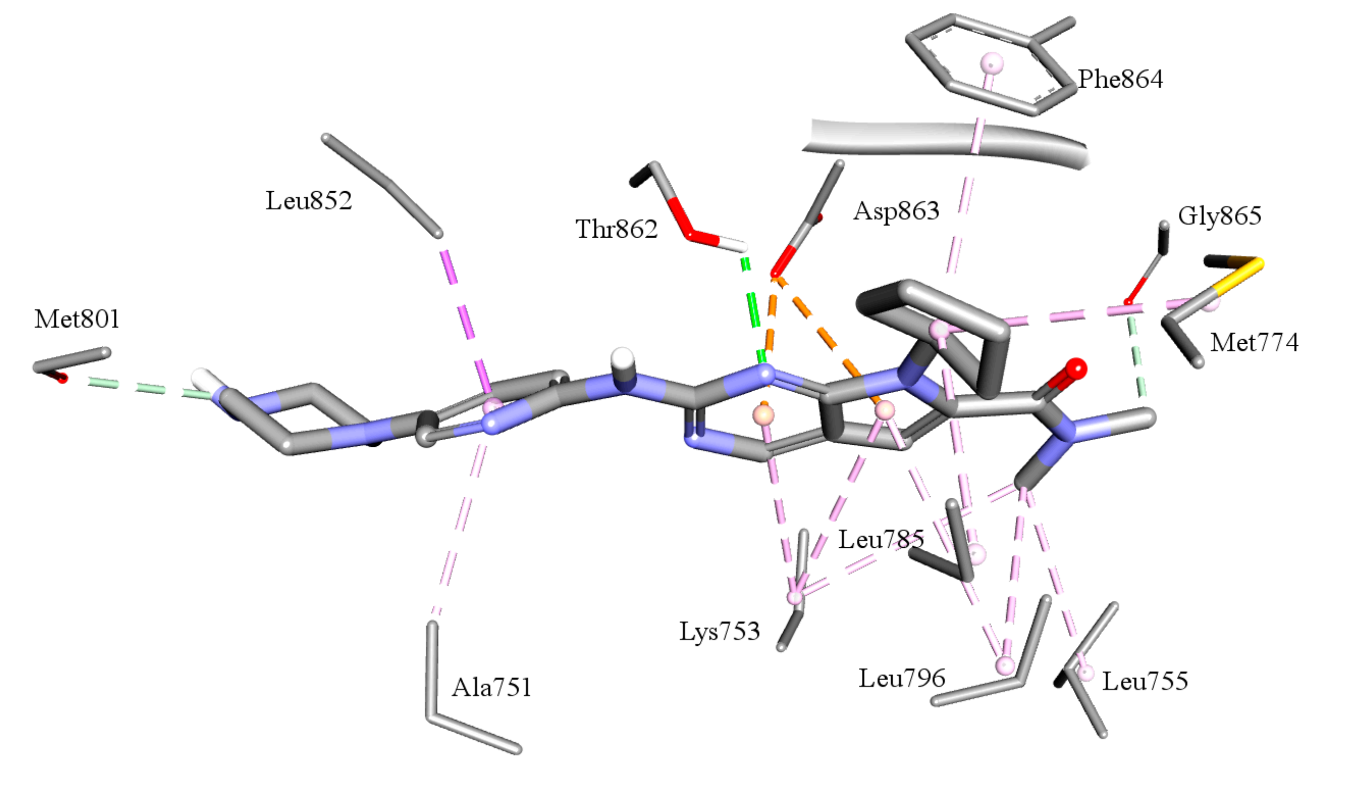

A

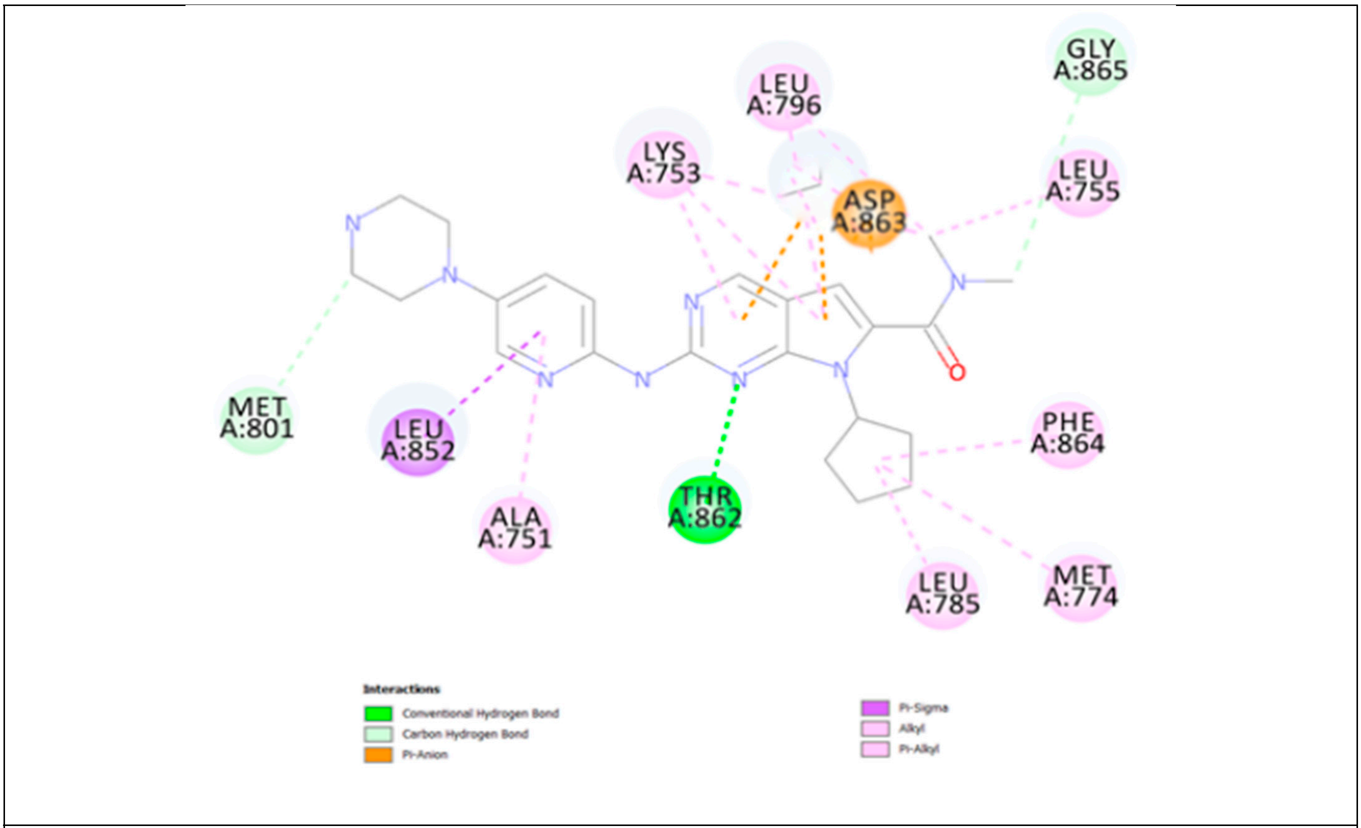

B

Figure S2: (A) Interaction forces of Ribociclib within the binding pocket of HER2 protein (PDB Code: 3RCD) (B) 2D presentation of Interaction forces of Ribociclib within the binding pocket of HER2 protein (PDB Code: 3RCD)
